# In/dependent Collaborations: Perceptions and Experiences of African Scientists in Transnational HIV Research

**DOI:** 10.1111/maq.12206

**Published:** 2015-05-14

**Authors:** Ferdinand Moyi Okwaro, P. W. Geissler

**Affiliations:** ^1^ Department of Archaeology and AnthropologyUniversity of Cambridge; ^2^ Department of Social AnthropologyUniversity in Oslo

**Keywords:** Africa, medical research, science studies, HIV, postcoloniality

## Abstract

This article examines collaboration in transnational medical research from the viewpoint of African scientists working in partnerships with northern counterparts. It draws on ethnographic fieldwork in an HIV laboratory of an East African state university, with additional data from interviews with scientists working in related research institutions. Collaboration is today the preferred framework for the mechanisms by which northern institutions support research in the south. The concept signals a shift away from the legacy of unequal (post‐) colonial power relations, although, amid persisting inequalities, the rhetorical emphasis on equality might actually hinder critical engagement with conflicts of interest and injustice. To collaborate, African scientists engage various strategies: They establish a qualified but flexible, non‐permanent workforce, diversify collaborators and research areas, source complementary funding to assemble infrastructures, and maintain prospective research populations to attract transnational clinical trials. Through this labor of collaboration, they sustain their institutions under prevailing conditions of scarcity.

## Introduction

Clinical trials increasingly occur on a global scale, as industry and governments in wealthy countries in the north move trials to poorer countries in the south. This process, sometimes referred to as globalization of clinical trials (Glickman et al. 2009; Parker and Bull 2009), has received attention from anthropologists because of their traditional geographical interests and attendant concerns with poor or vulnerable communities combined with the anthropological turn to science and technology (e.g., Cooper 2008; Petryna 2005; Rajan 2006; Simpson et al. 2009).

Like other anthropological studies of globalization or economic neoliberalization, this research attends to post‐imperial geographies intertwined with struggles of class and gender and emphasizes polarities of economic value and power, exploitation, and domination (see Rottenburg 2009), which also underlie bioethicists’ concerns with vulnerable study populations and with social value and justice. Such issues are obviously critical under conditions of radical inequality. And as important as objective material imbalances, so are experiences, reflections, and debates about injustice that arise for those working under these conditions and the resulting subjectivities and relations (Sleeboom‐Faulkner and Kumar 2011).

An issue that, though linked to actors’ subjectivity, intentions, and experience, has received comparatively less critical attention, is the will to do good, to do one's work well, and the desire to improve science, people's lives, and society (e.g., Prince 2013; Tousignant 2013a). Foregrounding this optimistic premise or promise of global health does not distract from questions of inequality and political–economic interest, but allows a more nuanced understanding of contradictions and possibilities. In his recent ethnography of MSF, for example, Redfield (2013) scrutinizes the landscapes of doing good, in particular the “double bind” of medical solidarity across boundaries of inequality (Redfield 2012). Rather than determined by the tectonics of political economy, political (im)possibilities derive here at the intersection of territory and action, and desire and affect. Similarly, Wendland's (2012) ethnography of African doctors’ ideals and hopes attends to longings and contradictions within a predictably bleak backdrop of resource‐deprived health systems and inequalities.

Inspired by these exemplars, our on‐going study of African scientists in international research takes collaboration or partnership among global scientists as its starting point. These promissory terms can obscure underlying inequalities and, in a narrow sense of the political, depoliticize transnational research (see Geissler 2013). Yet, maybe more importantly, in the hands of skilled actors, they provide the building materials and tools for diverse and innovative social and scientific practices and surprising possibilities (see Gerrets 2014).

## Making Global Health Collaborations in Africa

Our interest in the pursuit of good science through collaboration reflects regional peculiarities. Some economically deprived parts of the world—notably South Asia and some post‐socialist eastern European countries, which offer cheap medical professionals, viable infrastructure, and treatment‐naive populations—have seen an influx of private, for‐profit pharmaceutical trials, often of drugs destined for wealthier populations (Petryna 2005). In Africa, by contrast, the globalization of medical research affects especially *public* health research, funded mainly by not‐for‐profit bodies and are targeted at local health challenges (Geissler 2015; Prince 2013). This results from two complementary movements: vitiated nation‐state capacity and increased transnational investment and intervention. Throughout the past three decades, often shorthanded as the era of neliberalization (Ferguson 2009; Garrafa et al. 2010; Navarro 2007; Ong and Collier 2005), African governments have become increasingly unable to or unwilling to fund scientific research and health care (Ferguson 2006; Pfeifer and Chapman 2010).^1^ Northern governments, charities, and public–private consortia have simultaneously increased funding for research on diseases such as HIV/AIDS, malaria, and other neglected diseases—responding to global academic interests and local medical needs (e.g., Crane 2010).

The use of collaboration as a framework for the transnational mechanisms that support research activities in the countries in the south is relatively recent. It replaces earlier concepts such as, for example, the colonial expeditions and surveys of imperial science or post‐colonial transfers of funding and expertise premised on developmental solidarity (Gaillard 1994). This changing phraseology reflects a shift in the mechanisms of resource transfer. It also indicates a desire to detach current structures and practices from the legacy of colonial power relations (Crane 2010) and to avoid reproducing them through the invocation of an ethic of collaboration, which should entail a more balanced vision of control and responsibility. The resulting collaborations between African institutions and partners from the north have been hailed as beneficial for economic, scientific, moral, and political reasons (see e.g., Benatar 2002; Benatar and Singer 2010; Bhutta 2002; Costello and Zumla 2000; Jentsch and Pilley 2003; Parker and Bull 2009; Simpson et al. 2009; Wight 2008). Often used synonymously with partnership, collaborations are envisioned as predicated on the core moral values like freedom, autonomy, independence, and equality that pervade all levels of collaborative clinical trials: from participant‐informed consent, through peer group volunteering and staff contracting, to institutional and inter‐governmental collaborative agreements.

Yet, as Samoff and Carrol (2003) noted a decade ago, this terminological shift has not been accompanied by critical studies of collaborative practice (for an exception see, e.g., Agnandji et al. 2012). Imbued with positive moral value, collaboration is removed from analytical scrutiny. The role of especially anthropology in collaborative research sites is usually limited to understanding the community and eliminating barriers to participation in clinical trials: Hopes and anxieties that scientific collaborations engender among research participants and communities have thus been amply studied (e.g., Fairhead et al. 2006; Geissler 2005; Okello et al. 2012; Saethre and Stadler 2013; White 2000). The social in medical research is here about local lay people, largely excluding the world of science itself. The aspirations, fears, and anger of African scientists received little attention.

Contemporary discourses on transnational research—concerning funding mechanisms, managerial structures, and research ethics regulation—consider collaborations mostly as unproblematic and straightforwardly beneficial for both the wealthy sponsor (nation, institution, or charity) and the lower income host (for important exceptions, see Bradley 2008; Crane 2010). In reality, however, as evidenced by local public debates (see e.g., Fairhead et al. 2006; Geissler and Pool 2006; Patel 2006), transnational collaborations are open to diverse interpretations and harbor considerable potential for friction. A recent report in *Nature* suggests there may be tensions even in a model, world‐leading collaborative research program, where, apparently, African junior researchers instituted court proceedings against their management for what they referred to as scientific exploitation, even “modern day slavery” (Nordling 2012). Despite the shallow, journalistic nature of the article in question, this news item is of interest, partly because of the recognized excellence of research, capacity building, and community engagement at the collaborative site in question. More importantly, the case was widely discussed among scientists in south–east African countries, where the protagonists were sometimes referred to as the Kemri Six, with reference to the South African freedom heroes Sharpeville Six—suggesting a more widespread perception of tension with political and historical connotations, despite, or as part of, highly productive and mutually beneficial collaborative science across the region.

Scientific collaborations in developing countries bring together scientists and populations across wide disparities in education and experience, economic and social standing, and levels of health and health care provision (Glickman et al. 2009; Simpson et al. 2009). They are sustained—in many cases for decades—by continuous transfers of funds, expertise, and technological organization from the north, which has led critics to question whether the phraseology of collaboration might not, in fact, camouflage underlying asymmetry and northern dominance (Caffentzis 2002; Crane 2010; Gaillard 1994), and hinder open discussions and negotiations of the same (Geissler 2013).

Rather than being self‐evident and stable, collaboration is an encompassing and innovative social concept that is constructed, evolves, and takes varying shapes depending on the contexts and the groups involved. Collaborations require sustained effort to bring and hold together layers of interdependent actors—local and international scientists and their respective institutions, local scientists and their local collaborators, and research employees and trial participants. It is this work of transnational collaboration, in particular African scientists’ experiences and strategies, that interests us.

## Methodology

This article is based on ethnography from a research department at a prominent East African public university, which we identify by the acronym UHL (University HIV Lab). Responding to the shortage of ethnographic accounts of African scientists’ working situations and strategies (for exceptions, see, e.g., Feierman 2011; Langwick 2011; Tousignant 2013b), it focuses on African scientists, laboratory technicians, mobilizers, administrators, nurses, and counselors involved in medical research. We aim to present these science workers’ actions within a specific local context, rather than as mainly defined by transnational structures. Northern collaborators—scientists, administrators, students—feature, therefore, on the edge of the ethnographic field of vision. Our larger project will provide a more symmetrical ethnography of collaborative partnerships on a global scale.

Ethnographic techniques employed include in‐depth interviews, group discussions, informal chats, and observations with scientists and other staff. To obtain a wider range of senior scientists’ perspectives, we also conducted in‐depth interviews with scientists who previously worked at the UHL and with scientists from other research departments within the same university and from surrounding research institutions. We conducted 29 in‐depth interviews—20 with UHL employees and nine with researchers from other institutions—and had six focus group discussions with members of the community advisory board and peer leaders. In‐depth interviews and focus group discussions were conducted by Okwaro Ferdinand (OF) over five months of fieldwork; Wenzel Geissler (WG) joined in informal discussions with informants and visited the sites. Both authors have wide experience with ethnographic studies in the area and with medical research environments; both had previous professional relations with some key informants and institutions; and both worked together on study design and analysis.

OF grew up in East Africa and studied at the university. He could draw on personal knowledge of key events and debates during the formation of UHL, which was helpful to establish rapport and generate discussions about collaboration. As in all “anthropology at home,” previous familiarity with the site and some interlocutors was a potential source of bias and blind spots, but the advantages of trust and familiarity outweighed this risk. OF's status as a local scientist was, moreover, contradicted by the fact that his research was based at the London School of Hygiene and Tropical Medicine (LSHTM), a leading northern collaborator in global medical research—and funded by the Wellcome Trust, one of Europe's largest funders of collaborative tropical medical research in Africa. While carrying personal memories that enabled OF to empathize with the African university scientists, his project was at the same time of northern origins and a collaborative endeavor, making him both similar and different from the interlocutors. WG similarly had a double role as anthropologist of science in Africa and as northern collaborator and PI. The authors acknowledge their position, being within and without the flow of experiences that they set out to examine, and made it explicitly part of their own collaborative practice, their engagement with interlocutors.

Institutional ethnography is not without difficulties. Ethnography can be intrusive and demanding for busy researchers; inviting informants to discuss collaborative relations may threaten these relations. Furthermore, discussing collaborations with different staff at one department implies that junior staff discussed what essentially was their seniors’ domain. Notwithstanding anthropologists’ attachment to participant observation, the researcher's presence and daily interactions with employees, including senior managers, might, in fact, have occasionally hindered more candid discussions of what interlocutors considered sensitive issues. Occasionally, junior staff redirected such aspects of collaborative engagements to their seniors: “On that one you can get the details from management.” Yet, senior staff, too, was at times understandably guarded and unwilling to share confidential information, especially because the researcher discussed the same topics with junior staff. We therefore decided to include one‐off in‐depth interviews with scientists from other departments and institutions, which were less ethnographically entangled with the researchers.

Ethical review for this project was obtained from LSHTM's ethical review board and the local ethical review board of the studied institution. Employees were informed about the research by their management through email; all interviewed respondents provided individual informed consent.

## The Research Site

UHL, a research department at a major teaching and referral hospital in East Africa, was established in early 2000, when global hopes for an HIV vaccine ran high. Advances in vaccine development, combined with related questions about genetic and antigenic variability of HIV, necessitated the search for trial sites outside Europe and North America (Esparza et al. 2002; Streefland 2003). UHL was established with a skeleton staff of three (a clinical trial physician, a laboratory technician, and a secretary) to spearhead HIV clinical trials, initially receiving infrastructural funding from one single collaborator, an international consortium of funders and scientific institutions, who also provided the inaugural vaccine candidate. UHL's current director recalled:

I was writing my post‐doc at [X^2^] when one of the promising HIV vaccine candidates was developed at this university and the people were looking for a group that would move the vaccine trials to humans. We realised then that the most logical approach was to take the vaccine to [Y^3^]. There was then the big issue; we in Y had never done this thing before. How were we going to manage it? On that one I said, “Why don't you train me?” So between 1999 and 2001, we were putting the infrastructure together, human resources, research communities, developing our labs, and in 2002 we carried out the first human vaccine trials.

Since its foundation in 2000, UHL has developed into an exemplary research center, employing over 56 staff in two study sites—one on the university hospital campus, and the other at a council clinic in a nearby informal settlement—complete with community advisory boards and peer leaders who engage the communities from which trial volunteers are recruited. Since its inception, UHL has conducted many studies; attempts to establish the exact number were waved away by UHL's deputy director: “They are many—we only keep a record of those that we concluded successfully. The rest we do not record.”

UHL is both within as well as, in important ways, outside the university. It is accountable to the university's legal, financial, and administrative structure, and all collaborative agreements are signed by the university's administration, which thus holds legal responsibility. UHL is required to comply with university regulations such as the procurement procedures—a condition that sometimes raised issues with collaborators. As UHL's deputy director observed: “A collaborator does not understand how in a country with huge unemployment, it takes us over six months to hire a nurse or buy a car. They want that if they give the money today, we should have the nurse or the car by next week!”

For its day‐to‐day operations, however, UHL operates independently, headed by a director and a deputy assisted by a team of five administrative and financial managers. All salaries are paid from collaborative funding. Only the director and deputy director hold university positions and receive permanent, if modest, salaries that they complement with allowances, per diems, and other payments related to projects, which have become an expected part of collaborative transfers (see Ridde 2010; Vian et al. 2012).

Although UHL has the largest budget of all university departments, due to the time‐limited nature of donor funding, most of its employees can only be employed on short‐term, pension‐less, though comparatively well‐paid, contracts that are renewed every year. At the same time, there is a huge turnover of staff in between projects. One of the leading research scientists and trials managers at UHL, employed on one‐year renewable contracts for over 11 years, commented: “We are always firing and hiring. We can dismiss a nurse today, only to advertise for the same position in a month's time.”

## Perceptions and Experiences of Collaboration

Most interviewed scientists concurred that biomedical research in their institutions would be impossible without northern collaborators. Government support was usually limited to basic salaries for key staff and elementary facilities for research, while scientists had to solicit external funds for research. A highly trained and motivated non‐UHL scientist thus lamented: “Without donor funding, I would come to the lab, read the newspapers and go back home in the evening”—a sorry fate encountered in other research institutions and university departments that were not able to attract collaborators. At the onset of this study, UHL's director remarked: “Everything you see here has been obtained from donor funding,” not so much admitting to the inherent dependence on northern partners as underlining a shrewd ability to attract funding.

Scientists from different institutions and disciplines provided varying opinions on what they perceived to be the nature of collaboration. Within UHL, senior scientists and staff at the management level projected a picture of equality and autonomy and of seamless collaborative operations with structures that forestalled friction or resolved disagreements. When pressed to give examples of disagreements, UHL's director waved it off: “It is like in any marriage, friction is to be expected but we always find a common ground and move on,” while the deputy director commented “that (friction) only occurs at the beginning or with new partners.” Even these comments were proffered quite reluctantly in response to prodding about a conflict between UHL and one of its first collaborators, which had received huge media coverage. Scientists at the management level also underlined their independence by citing collaborations they had rejected. UHL's deputy director thus expressed reluctance to team up with a new collaborator whose demands were unreasonable:

When working with other collaborators we only need to show them that we have a GCP [good clinical practice] certificate and that suffices, but this company wants to retrain us, just so that we can have a certificate with their name on it. So here you are, with your own certificate, why should you take 10 hours to get one that has their name? Some of their demands can be very unreasonable, but they are a private company and those are their rules and so we have to comply.

Though told to underline instances of independent judgment, the anecdote concedes that, ultimately, UHL must comply. Clarifications of this nature were common in discussions with management‐level scientists who were eager to marry the textbook vision of collaborations with the complexities of its reality. To demonstrate their expertise and their equality with their northern counterparts, they often listed research projects in which they were principal investigators, even for projects designed exclusively by their collaborators prior to assigning what has come to be called a “local PI.” As one of the management scientists observed, “A good collaboration is where we are also *allowed to be* principal investigators” (emphasis added). As in this case, even the most confident praise of good collaboration could not avoid referencing power differentials. But this was done in an off‐hand, deflecting manner, not by drawing explicit critical attention to it—at least not by the lead UHL scientists.

In contrast to these positions provided by the most senior UHL scientists, other UHL staff members—junior scientists, technicians, counselors, mobilizers, and field staff—were significantly more critical of collaborations, sometimes contradicting their own management. Most of them thought that collaborations were starkly unequal and that this problem required some rethinking and reconstitution. One particularly contentious issue in this regard was the distribution of resources and duties across the collaborative network. Using the example of income differentials, one leading scientist at UHL wondered why for some aspects of science universal standards applied, while for others, notably scientists and technicians’ remuneration, local standards were used:

I do not know where that came from, but the idea that universal standards should be maintained in scientific procedures but not for remuneration is not fair. If we are downgrading the salary to local standards, then why not also downgrade the quality of the science to local standards? We would, then, say, “You know this is a poor country and so if their refrigerators are not good and the specimen thaw a bit, that is okay, those are the local standards. If the lab technicians are poorly remunerated and mess up the experiments a bit, that is okay, those are local standards.” Another thing; why do the funders assume that the local standards are fair and everyone is happy with them?

Another area of discontent concerned the question of decision‐making in the research process, as another UHL scientist, also employed on temporary contracts, points out:

The way I see it is that our collaborators control everything and when our definitions and theirs differ, their opinions carry the day. Recently they terminated a study because they said we did not recruit the right type of volunteers. We recruited high risk volunteers as required by the study protocol and our collaborators expected a number of them to become infected within the first six months of the study. When they did not, they concluded that we had not recruited the correct group and terminated the study after only six months.

Even more openly critical views on collaborations came from former UHL scientists and scientists in other research institutions. A leading former UHL scientist was critical of research collaborations in general and those at UHL in particular: “At that time when I worked at UHL, when we disagreed with our partners, they were quick to point out that they had ‘only hired us’—we were never their partners; and (after a long pause he continued), I don't think anything has changed since then.” This scientist had been at UHL when UHL disagreed with one northern collaborator about patent rights of a promising HIV candidate vaccine—a dispute that was widely reported in the media at the time and that UHL's current director acknowledged as “teething problems to be expected at the beginning of any process.” Related to this case, a prominent scientist from another research institution stressed the importance of clarifying the nature of relationships from the onset to guard against future disagreements: “Any time they come to me asking for collaboration, I always ask them: ‘Do you want me to work with you or to work for you?’”

Another former UHL scientist who had established a different but equally high profile research unit within the same university, also sustained solely by collaborative funding, was candid about the inevitably unequal nature of collaborations:

Collaborations do not necessarily imply 50/50% stake in everything, even an 80/20% is acceptable—for example, when we have 10 scholarships on a project, we know eight will go to candidates from our collaborator's country while we shall get two, that is normal. Even in your own family, you first take care of your own children before your neighbors’. In terms of contributions, they give me the money and I get them the samples. That is how it works.

For this scientist, collaborations did not necessarily imply equality but the provision of a complementary effort and an acknowledgment of the same. This stoic view might be referred to as “normal inequality,” to borrow from Feierman's (2011) assertion of health conditions in Africa as “normal emergencies.” A similar position regarding collaborations was shared by a few other scientists, although they were not as direct in their categorizations. One leading scientist objected to the use of the term “collaborations” itself: “Can we really refer to these as collaborations?,” suggesting that the term was applied to institutional forms that were not altogether different from earlier unequal modes of conducting science in Africa, echoing the critique of “neo‐colonial” science sometimes found in the literature (e.g., Boshoff 2009).

Different scientists held different opinions regarding the nature of collaborations, some emphasizing their smooth operations and the independence of the partners, others pointing at conflicts and critiquing dependency. But even those who insisted that collaborations worked well drew attention—albeit sometimes inadvertently—to inequalities and dependencies. One possible explanation for different views might be that informants worked with different collaborators with dissimilar modes of engagement. Yet, for the UHL scientists, who had all worked with the same collaborator(s), differences seemed related to their position: Interviews with researchers at management level yielded different views and opinions from those at lower levels, with the latter being more critical.

One would obviously expect senior researchers to have a more long‐term, cumulative, and therefore overall more balanced view of collaborations. However, the details of tensions and conflicts provided by technical staff lend some credibility to their view that collaborations indeed were conflictual. Another explanation for senior staffs’ positive portrayal of collaborations might thus be found in the performative skills of senior scientists, well versed in collaborative strategies that require some amount of lip service to the aims of partnerships and equality. Carrying the responsibility for maintaining collaborations, claims to equality were also vital for maintaining their sense of dignity and personal integrity. The lower ranking staff, though, working on the basis of short‐term contracts and particular job descriptions, do not seem to have this desire or responsibility to enact collaboration as balanced or frictionless. Moreover, senior staff negotiating collaborations over long periods of time may have seen them improve through what they considered as reasonable and dignified compromises. They were able to entertain more long‐term visions for their institutions, as well as their own careers. For them, equal collaboration was as much a way of speaking about an imperfect present as a tool to lay claims and pry open opportunities and promise for the future. Last but not least, they were paid better, presented at conferences and co‐authored publications, and were thus better placed to experience “equality.”

This diversity of stated experiences and perceptions is itself an important aspect of collaboration, as it contradicts any simplistic representation of transnational collaborations in terms of exploitation or domination and points us to the strategies that scientists adopt to operate them effectively. The remainder of this article will examine these strategies.

## Making Collaborations

Collaborations were, according to our informants, initiated in three ways. One was by a long‐standing association with a northern institution that assisted in the African partner's initial formation. UHL's initial research had come about in this way, but had somewhat diversified since (see below). In the collaborative sites outside the university, however, scientists relied on one initial, large‐scale main funder from Europe or North America. In these cases, the African research institution acts more or less as an outsourced station for research work by the northern partner, as sometimes reflected in the names given to collaborative entities, such as “field station” or “X unit,” referencing enduring attachments to the main donor. One UHL scientist summarized: “This is how I see it, we collaborate with Y in that we provide the sites for testing the vaccines, we have the personnel and the lab, which have all been established with the support of Y, and in most cases Y has actually initiated the work.”

Overall, this mode of collaboration was seen as problematic, not least because it entailed a potential for the visiting partner to become an effective gatekeeper, controlling access to staff, laboratory facilities, and even study populations. In such cases, the African scientists strived to establish additional collaborations to counterbalance this dependency. As one senior non‐UHL scientist suggested, such monolithic overseas‐led sites “have had their time.” While beneficial during a time of scarce resources and expertise before the turn of the century, the single mega‐collaborators have become a limiting factor for a dynamic and skilled generation of African scientists.

A second way of establishing collaborations was when African scientists responded to calls from northern funders. Since these calls often required the formation of collaborations between north and south, African scientists must purposely find northern partners—chosen on account of their scientific standing and links with funders as well as previous experiences with them. Most scientists preferred this method of initiating collaborations because they could be proactive; as a result, rights and responsibilities of each partner were spelled out in project proposals and clinical trial agreements. As one UHL scientist explained: “We enter into the research as *equals* and solicit for funding from one donor. This way no one feels like he is superior to the other” (emphasis added).

A third way in which collaborations were initiated was by the reverse process whereby northern scientists and institutions sought southern collaborators, which increasingly is a political requirement by funders. Southern partners in this type of collaboration are chosen by their location, access to the field, qualified staff, and track record of accountability and efficiency. Many of the interviewed scientists described collaborations initiated in this manner as fraught with friction and posing the greatest risk for exploitation, as a UHL scientist said: “Here, when you disagree in the process, someone says, ‘You know I only co‐opted you to fulfill the requirement from my donors that I have to collaborate with a scientist and institution from the south.’” In this scenario, there is a tendency for the terms of reference to be set by the northern partner and stoically accepted as an inevitable part of making a living in cash‐strapped African institutions (see also Bradley 2007).

In practice, the interviewed scientists engaged in all three types of initiating collaborations, although most preferred the second, and valued diversity of collaborative modes. The scientists at UHL operated from a comparatively open platform, partly because their main funder was not providing ever‐expanding collaborative support. Senior UHL staff emphasized the resulting freedom to choose collaborators. Yet, several non‐ and ex‐UHL scientists challenged this self‐representation, claiming that initial funders (in UHL and other collaborative sites) often acted as gatekeepers. One ex‐UHL scientist was categorical, “You cannot work in UHL without going through university [K^4^] and everybody knows that you must obtain permission from [Q^5^] to work in [Z^6^].”

## Infrastructure, Technical Capacity, and Gate Keeping

In the science worker's reflections about independence and control in collaborations, technical infrastructure and capacity play crucial roles. In one prominent strand of discussion, availability of local technical capacity is linked to questions about ownership of biological specimens and where these are to be analyzed—and thus to the question of who owns research. Lack of local capacity—in terms of equipment and training—can require exporting samples; conversely, the demand to analyze samples locally may necessitate the transfer of apparatus or skills. Scientists strongly associated the capacity to conduct analyses locally with equality and autonomy, and characterized collaborations that involved shipping of samples as short‐lived and thus undesirable. UHL's director explained: “These are sometimes referred to as ‘parachute researchers’ because they come with a parachute, pick the samples and go with a helicopter, very fast. And once this happens, the collaboration dies naturally.”

Comparing the present situation with a recent past, many UHL scientists stressed that they now had the capacity to conduct all kinds of analyses and that they nowadays rarely shipped samples except for quality control. Yet, other scientists, two of whom had previously worked for UHL, disagreed, stating that many collaborators still preferred exporting samples and only invested in local capacity building for research that absolutely had to be conducted on fresh samples. The former UHL scientist who founded a new research institute, cited above, was outspoken (though seemingly unconcerned) about this: “Some of the equipment is so expensive to acquire and maintain, and so where experiments do not need to be conducted on fresh blood, why would donors install equipment on site?” Another former UHL scientist explained: “In biomedical research, samples are like gold, and no researcher will sponsor a study and leave without the samples.”

This debate about specimen export and local analytical capacity highlights African scientists’ key concerns and intentions (see also Tousignant 2013b). Yet, while infrastructural capacity is an obvious key to independent science, the continuous evolution of transnational regulations and standards,^7^ intertwined with the ever‐accelerating turnover of technical apparatus and methodological innovation, makes the acquisition and maintenance of an up‐to‐date infrastructure progressively demanding. While 20 years ago qualified technical staff could establish transnational collaborative science if they had a vehicle, a microscope, a faucet, and a plug, today's collaborative laboratories are certified by the International Organization for Standardization (ISO), maintained by international service contractors, reliant on patented reagents, and their apparatus is outdated within few years.

In recent years, massive external investment in local analytical infrastructure and data connectivity insidiously changed the conventional wisdom, according to which Africa‐based technical capacity equals freedom (in contrast to sample export as evidence of dependency). Large single collaborators, providing funding that exceeds the host nation's national funding for medical research, are able to set up costly world‐standard equipment (e.g., ISO‐accredited level‐3 labs and sequencing facilities) and to maintain and adapt it to rapidly changing technical and regulatory standards. Such potent collaborators tend to effectively control major research sites, including future collaborations with third parties; they thus end up becoming the local collaborator in their own right. Faced with this potential—reflected in a senior non‐UHL scientist's remark: “Where would a new collaborator go to ask for access? To the owner of the state‐of‐the‐art laboratory!”—the conventional wisdom of equating local laboratory capacity with scientific independence might need some rethinking.

## Strategies for Collaboration

Acknowledging the need to collaborate and its challenges, scientists developed strategies to constitute more “equitable” collaborations, obtain resources, and achieve some sustainability, creatively operationalizing the logic of collaborations. These strategies include diversification of collaborators and the scope of research, complementary funding, and positioning “wonderful research sites.”

### Diversification

Faced with diminishing funding from initial collaborators and lack of government support, scientists seek projects from multiple collaborators, in addition to the original donor. Such projects are initiated in different ways and come with varying levels and types of support, expectations, and requirements. Collaborators are held at a carefully maintained distance from each other and where required, funds, equipment, space and employees are earmarked for different collaborators. As the deputy director of UHL observed: “We keep separate files for each collaborator so as to avoid passing the expectations of one to the other.”

The collaborating African institutions did not seem to have standard conditions for new collaborations, but minimum requirements, which could be gleaned from discussions of projects that were rejected, as this quote from the UHL director shows: “We refused to collaborate with [X] because they wanted us to underpay our staff, and also [Y] because they wanted us to ship all the samples to them.” Even as this was a demonstration of flexibility in engagement, UHL's director admitted that they occasionally accepted collaborations where they shipped samples because “we do not understand the technology involved.” Or they agreed to collaborations in which they played second fiddle because they were not involved in their conceptualization. Conditions for or against collaborations were thus evaluated on a case‐by‐case basis.

### Scope

Diversification of the scope of research is another strategy. UHL's director, acknowledging the economic origins of this approach, compared UHL to a local fast food restaurant: “It is like, if you go to Afrobroiler restaurant, you will find them serving hamburgers, beef, sausages, etc. Nobody recalls that this restaurant actually got the name Afrobroiler because it initially served only chicken and chips.” Similarly, UHL had broadened its scope beyond HIV vaccine trials to include other drug trials, research into other communicable diseases, and even non‐communicable diseases, such as cardiac diseases, cancers, and diabetes, and, during this fieldwork, even negotiating collaboration with a psychiatrist. Diversification—even into areas beyond the actual scholarly capacity of the involved scientists—generates new funding, enables more efficient use of resources and capacities, and increases the institution's research profile, which was an all‐important aspect in competing for new collaborators.

### Complementary Resources

The crucial advantage of collaborative diversification for the African scientists, apart from providing a somewhat steady flow of funding, which allowed a modicum of independent decision‐making, was that they could make optimal use of very diverse opportunities—maximizing scientific possibilities and gain—and use different collaborators in a complementary fashion, putting together a functioning scientific institution from different bits and pieces. Scientific equipment was always sourced from an array of partners funding different aspects of research and equipment. As UHL's deputy director commented:

When one collaborator funds one aspect of the project, you look for another who funds the other instead of asking the same collaborator to increase the funding. The European Union, for example, does not allow you to buy computers or printers from their money so if you need computers, you check with another collaborator.

Only research institutions with a developed scientific infrastructure attract funding and are able to competitively respond to calls and invitations for collaboration. Developing infrastructure is thus like joining an elite club of institutions that can continuously conduct research. The converse scenario is what one observes in non‐collaborating research departments, where institutions and scientists lack infrastructure and are therefore unable to compete for collaborators, resulting in a downward spiral of research inactivity and decay.

Self‐presentation in a thriving scientific institution was dependent on certain key aspects of equipment such as functioning buildings and water, power, and communication supplies, photocopiers, and reliable transport. Yet it was not always possible to obtain these objects of basic equipment from northern funders—who often excluded infrastructure from their funding, presumably on the assumption that their partners already existed as viable entities. No less important than such infrastructure was outward appearance. Up‐to‐date and clean offices and furnishing, busy workspaces, signposting, stationery, and business cards as well as self‐confident key staff were crucial components in maintaining the impression of a promising and reliable collaborating institution providing “wonderful research sites.”

### Wonderful Research Sites

In the absence of government funding and in view of the competition for limited international funding, the ability to provide study sites and populations is an important asset. A leading UHL scientist described why collaborators pick out UHL over others: “They come here and find that we have this wonderful research site able to conduct any type of research.” While maintaining technical facilities is a key challenge, the other critical dimension of wonderful collaborative sites is the communities from which volunteers are enrolled for different types of trials. This is no less challenging and costly to create and keep up, and no less valuable. This human and somatic dimension of clinical research resources is equally affected by growing standardization and regulation, as is the mechanical apparatus. As the anthropologist Petryna (2009) notes, meeting ethical requirements and the recruitment and maintenance of research participants is one of the most time‐consuming and expensive aspect of biomedical research—and it, too, needs special, changeable expertise.

Again, this challenge has increased over the last 20 years: While up to the early 1990s in most African societies research participants could be recruited with the permission of medical authorities or local chiefs or teachers, today participants undergo extensive individual information and consenting procedures, which require an infrastructure of community engagement, information and consenting, document handling, and storage. Moreover, standards of GCP require better follow‐up of adverse events than in the past, which requires better control of participant populations, availability of clinical expertise, and access to health facilities and drug procurement. In addition, funders and collaborators, when initiating large research projects, increasingly demand beforehand documentation of a site's ability to recruit suitable participants of a given specification, within a specified period. In particular, prestigious competitively enrolling multi‐site clinical trials of novel vaccines and interventions commonly set site‐specific recruitment targets for particular kinds of people, such as those not affected by or highly susceptible to a particular disease, are treatment naive, are of a particular age and gender, or are in a sexual relation of a particular type. This again requires close demographic and health monitoring of prospective recruitment populations in advance of actual trials.

UHL invested considerable resources to maintain potential study populations. It used its mobilizers, assisted by selected members of the community, to disseminate relevant information, mobilize, recruit, and retain volunteers. Like the clinical trial volunteers themselves, such community volunteers, although essential for the maintenance of study populations, were rarely paid for their services, except for transport reimbursement. This was valuable for UHL, which did not have funding to employ actual staff.

UHL has recently used an innovative strategy to ensure a steady flow of trial volunteers that it internally referred to as “banking.” UHL continuously prescreened, counseled, and checked the HIV sero‐status of prospective trials participants within a particular area. These potential trial volunteers maintain the set of health and demographic qualities that qualify them for enrolment into prospective studies. Trial volunteers were, for instance, expected to use a reliable contraceptive provided by UHL, to take precautions against contracting HIV, and to be monitored periodically to ensure they still met the requirements for inclusion in upcoming studies. Such banking enabled a speedy recruitment process since enrollment into the study only required a reconfirmation of willingness and possession of characteristics that meet the requirements of the study to be conducted.

The maintenance of wonderful research sites entails social relations across a range of levels: with powerful global actors; with international academic institutions, big charities, and scientific funders, and their individual representatives with their particular personal interests and idiosyncrasies; and with poor, relatively powerless local research participants and communities, mediated through local volunteers or temporary workers. At each level of this collaborative trial community, there is an awareness of the collaborative nature of the undertaking—of the involvements between more powerful and less powerful actors and agencies both within African society and between different locations across the globe. This awareness of global collaboration raises expectations and hopes, and it may as well produce doubts and discontent.

## Collaboration as Aim

Collaborations in biomedical research are necessary and beneficial for scientists and institutions both from the north and the south. These partnerships, however occur, under conditions of gross economic and ethical inequality that serve both as a problem to be confronted and an opportunity for professional knowledge production. They are further compounded by dynamic and constantly evolving models of partnerships that both address and obscure inequalities, which nevertheless occasionally breed tension and conflicts. Adhering to the collaborative idiom, northern partners rarely address such inequalities openly, while—as we have seen above—their African colleagues quietly adopt strategies to deal with this asymmetry rather than confront it. There is thus, as others have argued, a need to find ways of speaking and acting about inequalities and diverging interests, as well as about one's shared commitment to equality, to make strides toward an ethic of truly equitable partnerships that brings on board the perspectives of both the African and northern partners (Aellah et al. 2015; Geissler and Okwaro 2014).

The vision of collaborative partnership that this article explored is, of course, a step forward from previous, more patronizing, colonial, and postcolonial modes of scientific cooperation, which engendered their own responses and resistances and which remain inscribed into existing transnational scientific relations. But at its best, collaboration is precisely that: a vision, an unfulfilled promise, an aim of balance that under prevailing conditions of radical imbalance requires persistent, targeted labor. And maybe, as a model for future social forms of science, it has indeed had its time, as the criticism of some of our interlocutors might suggest; maybe it has reached a point where it prevents change rather than orienting it; maybe it is time for a different promissory paradigm.

## Notes

1 The neoliberal “structural adjustment” policies of the 1980s (Caffentzis 2002; Ferguson 2009; Samoff and Carol 2003) cut government expenditure and prioritized basic over higher education, depriving universities and teaching hospitals of resources for research.

2 A leading European university.

3 The university in which UHL is situated.

4 UHL's key collaborator.

5 A leading northern biomedical research funder.

6 A leading, highly respected research institution.

7 This is driven in particular by the U.S. Food and Drug Administration and similar arrangements such as ICH‐GCP (International Conference for the Harmonization of Technical Requirements—Good Clinical Practice).
